# miR-106b-5p contributes to the lung metastasis of breast cancer via targeting CNN1 and regulating Rho/ROCK1 pathway

**DOI:** 10.18632/aging.102719

**Published:** 2020-01-27

**Authors:** Zheng Wang, Tian-En Li, Mo Chen, Jun-Jie Pan, Kun-Wei Shen

**Affiliations:** 1 Comprehensive Breast Health Center, Ruijin Hospital, Shanghai Jiao Tong University School of Medicine, Shanghai 200025, China; 2 Cancer Metastasis Institute, Fudan University, Shanghai 200040, China; 3 Department of Surgery, Huashan Hospital, Fudan University, Shanghai 200040, China

**Keywords:** breast cancer, CNN1, miR-106b-5p, Rho/ROCK1 pathway

## Abstract

Objectives: Breast cancer has been the second most prevalent and fatal malignancy due to its frequent metastasis to other organs. We aim to study the effects of a key miRNA-mRNA signaling in breast cancer.

Results: CNN1 was identified as the key gene in breast cancer by the bioinformatics analysis, and the downregulation of CNN1 in breast cancer tissues and cell lines was observed. Upregulating CNN1 inhibited cell survival, migration, invasion, and adhesion, but enhanced cell apoptosis. miR-106b-5p not only bound to CNN1 mRNA 3’UTR, but also promoted lung metastasis *in vivo*. Besides, the miR-106b-5p mimic enhanced breast cancer canceration by targeting CNN1 and activating Rho/ROCK1 signaling pathway.

Conclusion: Overall, our results proved that miR-106b-5p promoted the metastasis of breast cancer by suppressing CNN1 and activating Rho/ROCK1 pathway.

Methods: Bioinformatics analysis was performed to select the key gene in breast cancer. The overexpression and knockdown of Calponin 1 (CNN1) in breast cancer cell lines were performed to conduct cell viability, migrating, invasion, proliferation, adhesion, and apoptosis experiments. To identify the role of miR-106b-5p and Rho/ROCK1 in CNN1-induced breast cancer, a dual-luciferase assay, tumor lung metastasis assay, transcript half-life assay, and Rho/ROCK1 inhibition assay were performed.

## INTRODUCTION

Breast cancer (BRCA) has been the second most prevalent and dreadful malignancy that primarily occurs among females, leading to over two million cases and 626,679 deaths in 2019 based on the TCGA cancer statistics. The local invasion and metastasis to distant organs like lung, liver, and brain were responsible for breast cancer-related deaths [1–3]. Exploring the molecular mechanism underlying BRCA development specifically the invasion and metastasis is critical to diagnose and remedy breast cancer.

In the past few decades, accumulating studies have proved that the epigenetic abnormities including aberrant miRNA expressions are associated with the development of breast cancer [4]. miRNAs are broadly expressed in all sorts of normal and diseased tissues. They regulate gene expression at the transcriptional level [5, 6]. Accumulating studies have proved the potential of miRNAs in both the diagnosis and therapeutics of breast cancer [7–9]. For instance, miR-196a was identified as an oncomir to promote cell growth in breast cancer through targeting UBE2C [10]. Li et al. reported that miR-29c participated in suppressing the progression of breast cancer by targeting DNMT3B [11]. Interestingly, miR-106b-5p has been widely studied in various human cancer in the last seven years. Specifically, miR-106b-5p was reported to be a promising lung metastasis marker [12], and breast cancer recurrence and progression [13]. Yet, the specific effect of miR-106b-5p on breast cancer cell canceration was not clear.

Calponin first confirmed in the chicken gizzard, could bind actin thin filaments as a striated muscle troponin T-like protein to regulate the smooth muscle contraction [14]. Calponin consists of three distinct mammalian isoforms: calponin 1 (CNN1), CNN 2, and CNN 3. They act as a pivotal role in cell metastasis, embryonic development, and prostate cancer progression [15–19]. CNN1 can inhibit actin-activated myosin ATPase and Ca^2+^ dependent mobility of actin [20, 21], therefore being identified as a key participant in stabilizing actin stress fibers. Recently, many studies have proved that CNN1 participates in multiple cancers. *In vivo*, the CNN1 knockout mice contributed to the inhibition of malignant melanoma cells invasion compared to the wild-type mice [22]. *In vitro*, CNN1 was a tumor suppressor gene that became an indicator of cell migration in hepatocellular carcinoma cells [23]. Similar to the effect of CNN1 on hepatocellular carcinoma cells, CNN1 expression was downregulated and also played a negative role in uterine leiomyosarcoma [24]. CNN2 was proved to be an oncogene in breast cancer [25]. Whether CNN1 is an oncogene or a tumor suppressor in BRCA remains to be revealed.

In this study, CNN1 was identified by bioinformatics analysis, which was associated with the prognostic and tumor stage. Then we observed that CNN1 expression was reduced in tumor tissues and cells. Forced overexpression of CNN1 retarded the canceration of BRCA cells. Besides, the miR-106b-5p and Rho/ROCK1 participated in the regulating process of CNN1 to BRCA cells.

## RESULTS

### CNN1 and STAT1 as the key genes in BRCA

**Figure 1:**
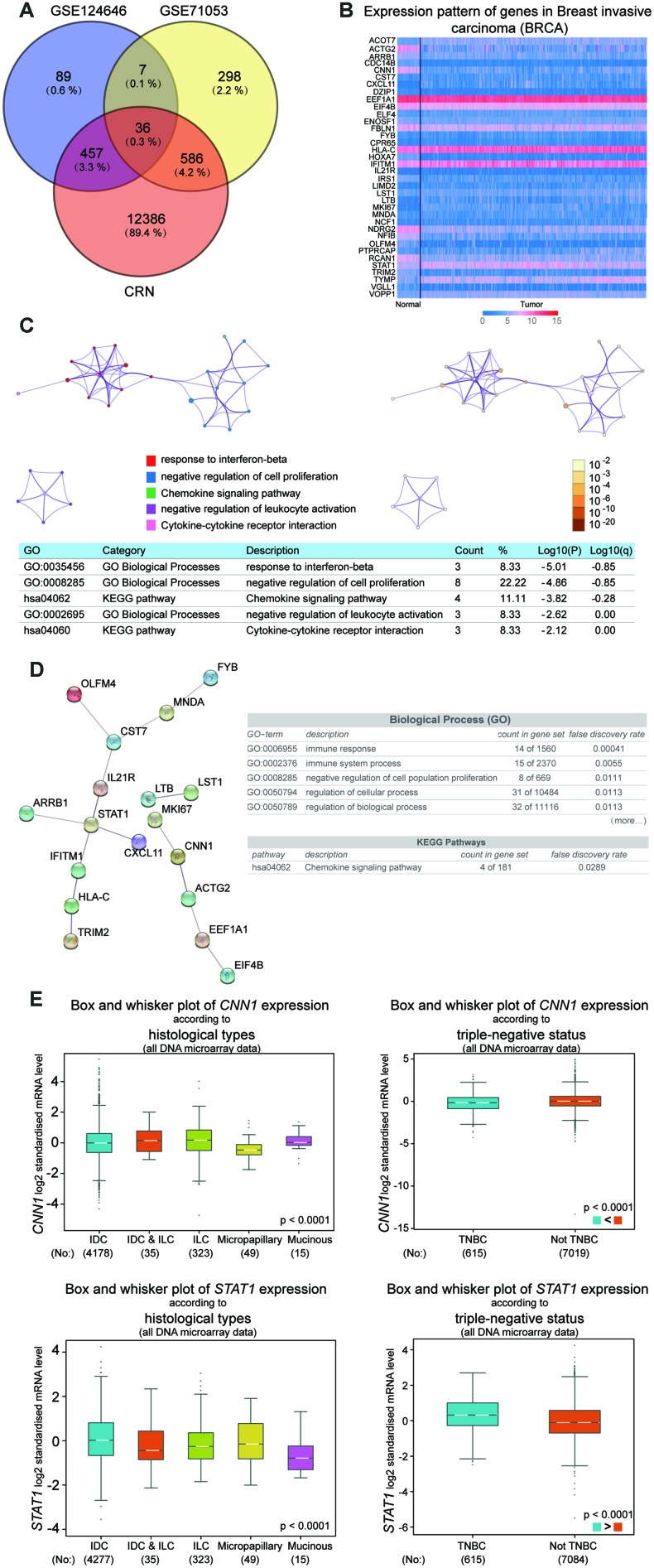
**CNN1 and STAT1 were the key genes in breast cancer.** (**A**) 36 common genes were screened after analysis with the results of the microarray chips. GSE124646 and GSE71053 were downloaded from NCBI. The DEGs of breast cancer was obtained from the Cancer RNA-Seq Nexus (CRN). (**B**) ACOT7, STAT1, TYMP, and VOPP1 were upregulated in breast cancer, while ACTG2, CNN1, CDC14B, NFIB, RCN1, and TRIM2 were downregulated in breast cancer. (**C**) The biological processes and KEGG pathway for 36 genes were analyzed using Metascape. (**D**) The String was performed to construct the PPI network, and analyze biological processes and KEGG pathway for 36 genes. (**E**) The expression of CNN1 and STAT1 according to different subtypes and TNBC status of breast cancer. Breast Cancer Gene-Expression Miner v4.4 was used to conduct the analysis. All DNA microarray data in the database were used. IDC, invasive ductal carcinoma. ILC, invasive lobular carcinoma. TNBC, triple negative breast cancer.

To reveal the key genes in BRCA, the microarray chip data of GSE124646 and GSE71053 were downloaded from GEO, and the DEGs profiles of breast cancer were downloaded from CRN. The DEGs was screened from GSE1244646 and GSE71053 with adj.P.Value<0.05. The 36 common genes were obtained by VENNT2.1 analysis (https://bioinfogp.cnb.csic.es/tools/venny/) (Figure 1A). Then the 36 common genes were uploaded to the UALCAN to analyze the expression level in BRCA. As shown in Figure 1B, the gene expressions of ACOT7, STAT1, TYMP, and VOPP1 were significantly increased in invasive breast carcinoma, while the gene expressions of ACTG2, CNN1, CDC14B, NFIB, RCN1, and TRIM2 were dramatically downregulated in BRCA. The Metascape and String overlapped the negative regulation of cell proliferation biological process, and the chemokine signaling pathway KEGG pathway (Figure 1C and 1D). Therefore, the CNN1 and STAT1 were identified as the common genes which were associated with the biological process and KEGG pathway. Breast Cancer Gene-Expression Miner v4.4 was used to further analyze the expression of CNN1 and STAT1 in different subtypes of breast cancer, indicating that the expression of CNN1 and STAT1 is markedly different in invasive ductal carcinoma (IDC), invasive lobular carcinoma (ILC), micropapillary, mucinous, and triple negative breast cancer (TNBC) (Figure 1E). We were particularly interested in IDC, TNBC as well as breast adenocarcinoma, which was not analysed by bc-GenExMiner. Finally, IDC cell lines (MCF-7 and T47D), TNBC cell line (MDA-MB-231), and breast adenocarcinoma cell line (CAMA-1) were chosen, and auxiliary qRT-PCR was conducted to detect the expression of CNN1 and STAT1 in those cell lines.

### CNN1 was crucial in BRCA

**Figure 2:**
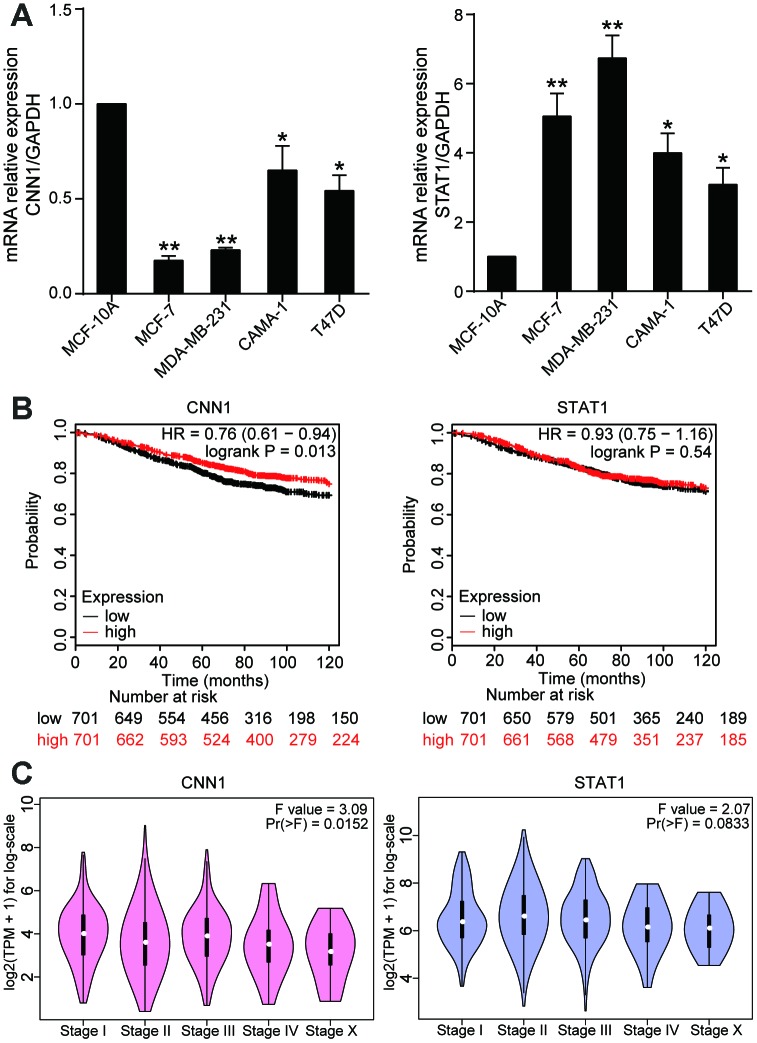
**CNN1 played a key role in BRCA.** (**A**) The mRNA expression of CNN1 was decreased in BRCA cells, while the mRNA expression of STAT1 was increased in BRCA cells. *P<0.05 vs. MCF-10A and **P<0.001 vs. MCF-10A. (**B**) The effects of CNN1 and STAT1 on the prognosis of breast cancer. (**C**) The effects of CNNA and STAT1 on the stage of breast cancer.

**Figure 3:**
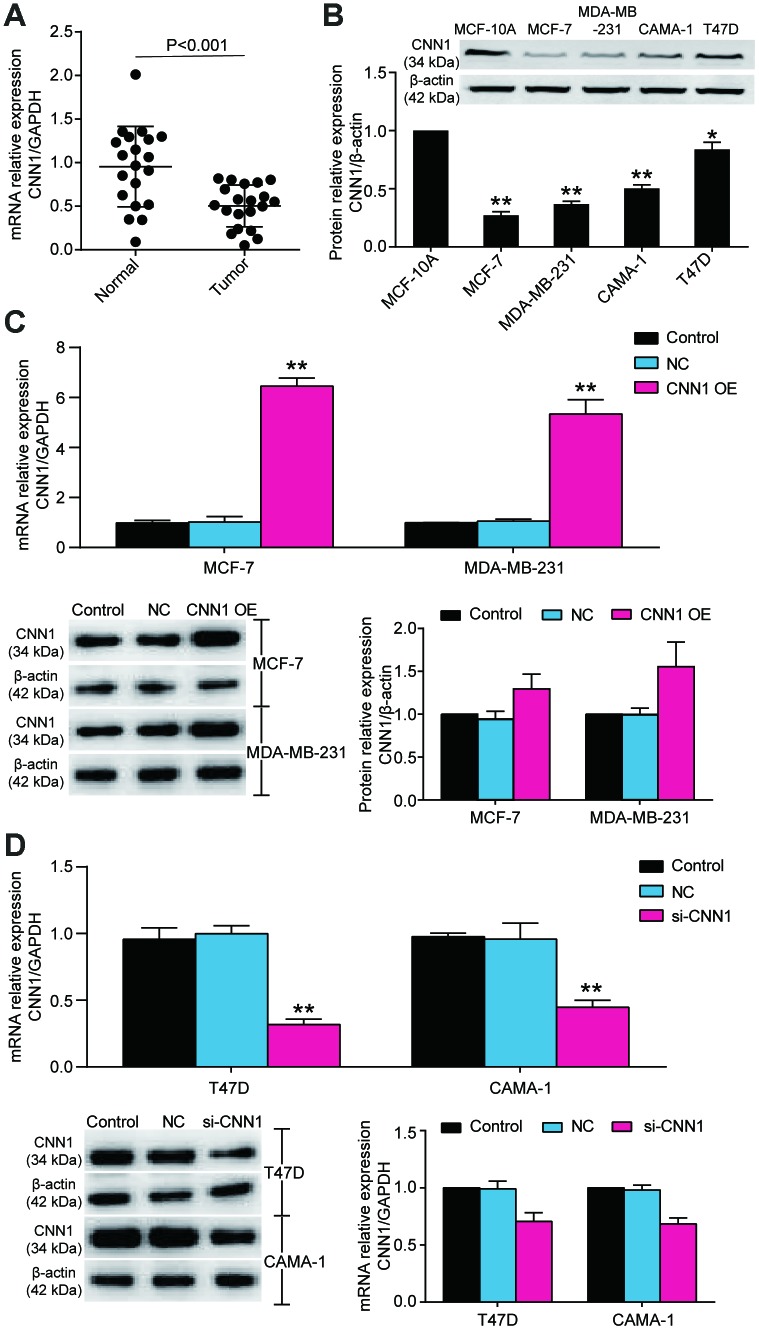
**The CNN1 expression was decreased in BRCA tissues and cells.** (**A**) The downregulation of CNN1 in breast cancer tissues (n=20) compared with normal breast tissues (n=20). (**B**) The protein expression of CNN1 was decreased in BRCA cell lines compared with the healthy breast cell line. *P<0.05 vs. MCF-10A and **P<0.001 vs. MCF-10A. (**C**) The CNN1 expression was upregulated after CNN1 overexpression transfected MCF-7 and MDA-MB-231 cells. Control, the cells were cultured without any treatment. NC, the cells were treated with the negative control. CNN1 OE, the cells were treated with CNN1 overexpression. *P<0.05 vs. Control and **P<0.001 vs. Control. (**D**) The CNN1 expression was downregulated after CNN1 small interfering RNA (siRNA) transfected T47D and CAMA-1 cells. Control, the cells were cultured without any treatment. NC, the cells were treated with the negative control. si-CNN1, the cells were transfected with CNN1 siRNA. *P<0.05 vs. Control and **P<0.001 vs. Control.

To identify the hub gene, we used qRT-PCR to measure the gene expressions of CNN1 and STAT1 in MCF-10A, MCF-7, MDA-MB-231, CAMA-1, and T47D cells. Compared with MCF-10A cell line, CNN1 expression was significantly reduced in BRCA cells (83% decrease in MCF-7 cells, 77% decrease in MDA-MB-231 cell line, 35% in CAMA-1 cell line, and 46% decrease in T47D cell line), while the STAT1 expression was dramatically upregulated in BRCA cell lines (5-fold increase in MCF-7 cells, 6.7-fold increase in MDA-MB-231 cells, 4-fold increase in CAMA-1 cells, 3-fold increase in T47D cells) (Figure 2A). The Kaplan-Meier analysis showed that CNN1 was a poor prognostic marker of BRCA (Figure 2B). Meanwhile, the CNN1 at the late stage of breast cancer was significantly decreased (Figure 2C). The Supplementary Table 1 displayed the interaction between CNN1 expression levels and the clinicopathological parameters of patients with BRCA. No significant correlation existed between CNN1 expression levels and age, tumor size, and histological grade in tissue samples owners. However, the expressions of CNN1 in Tis-T2 and T3-T4 of invasion depth were 0.63 and 0.32, respectively, with the significant difference. Similarly, a significant difference of CNN1 expression between N0 and N1-N3 was observed for lymph node metastasis (0.73 vs. 0.41). A remarkable difference in CNN1 expression was also observed for distant metastasis between M0 and M1 (7 vs. 13). Then we found that the expression level of CNN1 in BRCA tissues (n=20) was reduced by 50% (Figure3A), and the protein level of CNN1 in breast cancer cells was also downregulated, especially in MCF-7 cell line and MDA-MB-231 cell line (Figure3B). Our data demonstrated that CNN1 might act as a crucial role in BRCA compared with STAT1.

### CNN1 inhibited cell proliferation and invasion in BRCA cells

**Figure 4:**
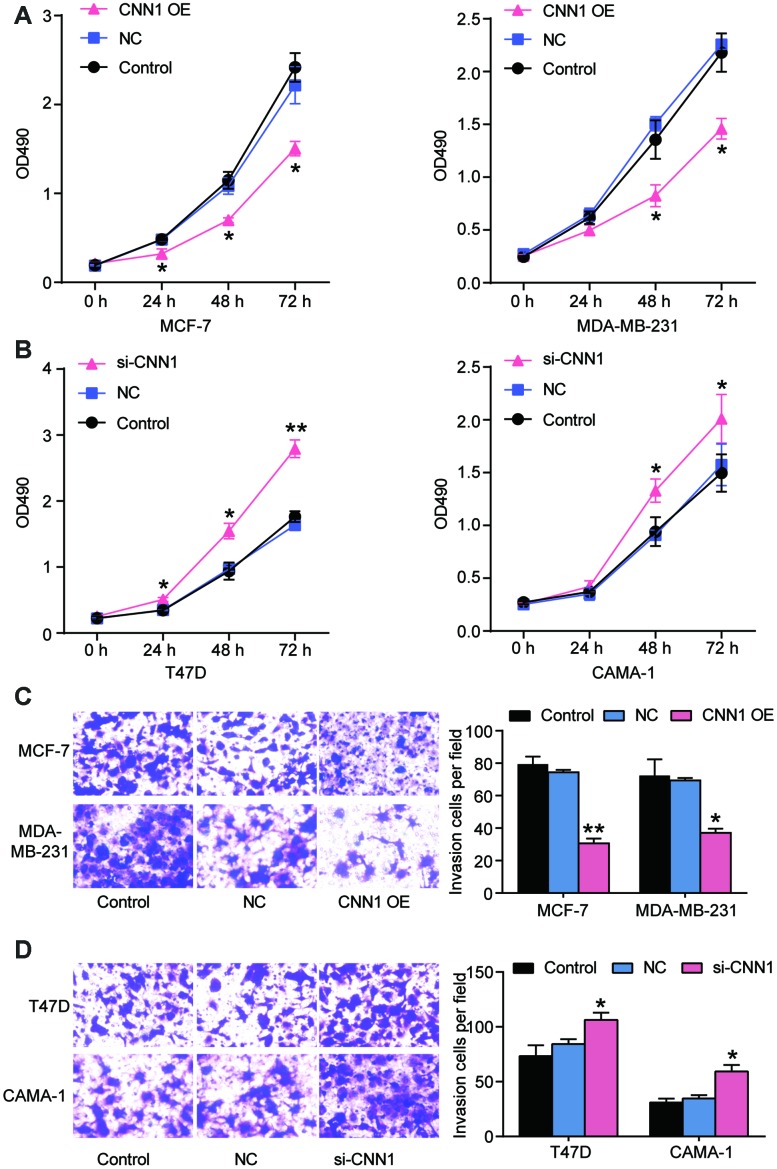
**CNN1 inhibited cell proliferation and migration of breast cancer.** (**A**) and (**B**) CNN1 overexpression inhibited cell proliferation in MCF-7 and MDA-MB-231 cells, while CNN1 knockdown promoted cell proliferation in T47D and CAMA-1 cells. CCK8 was performed to detect the ability of cell proliferation after the breast cancer cells (MCF-7, MDA-MB-231, T47D, and CAMA-1 cells) treated with negative control, CNN1 overexpression or CNN1 siRNA for 0 h, 24 h, 48 h, and 72 h. (**C**) and (**D**) CNN1 overexpression inhibited cell invasion in MCF-7 and MDA-MB-231 cells, while CNN1 knockdown promoted cell invasion in T47D and CAMA-1 cells. The cell invasion after CNN1 overexpression or CNN1 knockdown for 24 h was measured using transwell invasion assay. Control, the cells were cultured without any treatment. NC, the cells were treated with the negative control. CNN1 OE, the cells were treated with CNN1 overexpression. si-CNN1, the cells were transfected with CNN1 siRNA. *P<0.05 vs. Control and **P<0.001 vs. Control.

Due to the more significant downregulation of CNN1 in MCF-7 and MDA-MB-231 cell lines and less significant downregulation of CNN1 in CAMA-1 and T47D cell lines, MCF-7 cell line and MDA-MB-231 cell line were selected for CNN1 overexpression study, whilst T47D cell line and CAMA-1 cell line were chosen for CNN1 knockdown study. The mRNA and protein expression of CNN1 was successfully upregulated by CNN1 overexpression in MCF-7 and MDA-MB-231 cells, and the mRNA and protein expression of CNN1 was successfully downregulated by si-CNN1 transfection into T47D and CAMA-1 cells (Figure 3C, 3D). Meanwhile, the upregulation of CNN1 in MCF-7 cell line and MDA-MV-231 cell line effectively inhibited cell proliferation and invasion (Figure 4A and 4C), while CNN1 knockdown in T47D and CAMA-1 cells induced a significant increase of cell proliferation and invasion (Figure 4B and 4D). The results proved that the abilities of cell proliferation and invasion in BRCA cells were impaired via upregulating the CNN1 expression.

### CNN1 impaired the abilities of migration, colony formation, and adhesion, but enhanced the cell apoptosis rate

**Figure 5:**
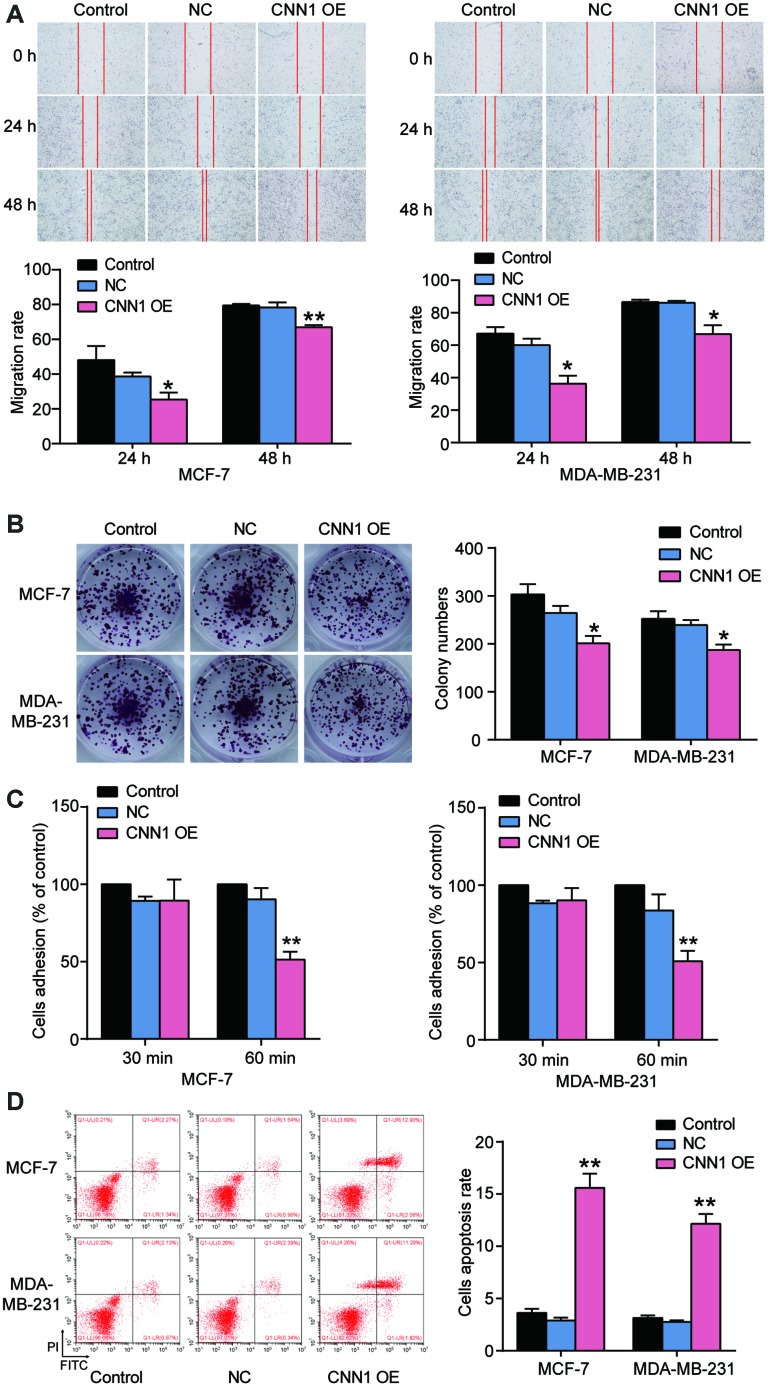
**CNN1 overexpression suppressed cell migration, colony formation, and cell adhesion, while CNN1 overexpression enhanced the abilities of cell apoptosis.** (**A**) The role of CNN1 overexpression in cell migration was confirmed using wound healing assay. The MCF-7 and MDA-MB-231 cells were treated with CNN1 overexpression for 0 h, 24 h, and 48 h. (**B**) The ability of colony formation was confirmed using colony formation assay. The MCF-7 and MDA-MB-231 cells were treated with negative control or CNN1 overexpression for 14 days. (**C**) The ability of cell adhesion after CNN1 overexpression for 30 min and 60 min was assessed using the cell adhesion assay. (**D**) The flow cytometry was performed to measure the cell apoptosis rate after CNN1 overexpression. Control, the cells were cultured without any treatment. NC, the cells were treated with the negative control. CNN1 OE, the cells were treated with CNN1 overexpression. *P<0.05 vs. Control and **P<0.001 vs. Control.

To reveal the role of CNN1 in BRCA cells, the wound healing assay and colony information assay were performed to measure the cell migration rate and colony formation. As shown in Figure 5A, the migration rate of CNN1 overexpression at 24 h and 48 h was respectively reduced by approximatively 80% and 30% in MCF-7 cells compared with control cells, and the similar effect of CNN1 overexpression on MDA-MB-231 cells occurred. The colony numbers of CNN1 overexpression were decreased to 33.7% of the control cells in MCF-7 cells, and 25.8% of the control cells in MDA-MB-231 cells, respectively (Figure 5B). Cell adhesion was significantly impaired at 60 min (Figure 5C) whilst cell apoptosis was promoted when forced overexpression of CNN1 was imposed on the cells (Figure 5D). These results confirmed that CNN1 overexpression inhibited cell migration, colony formation, and cell adhesion, but it could reinforce the abilities of cell apoptosis.

### CNN1 impaired the activation of the Rho/ROCK1 pathway in BRCA cells

**Figure 6:**
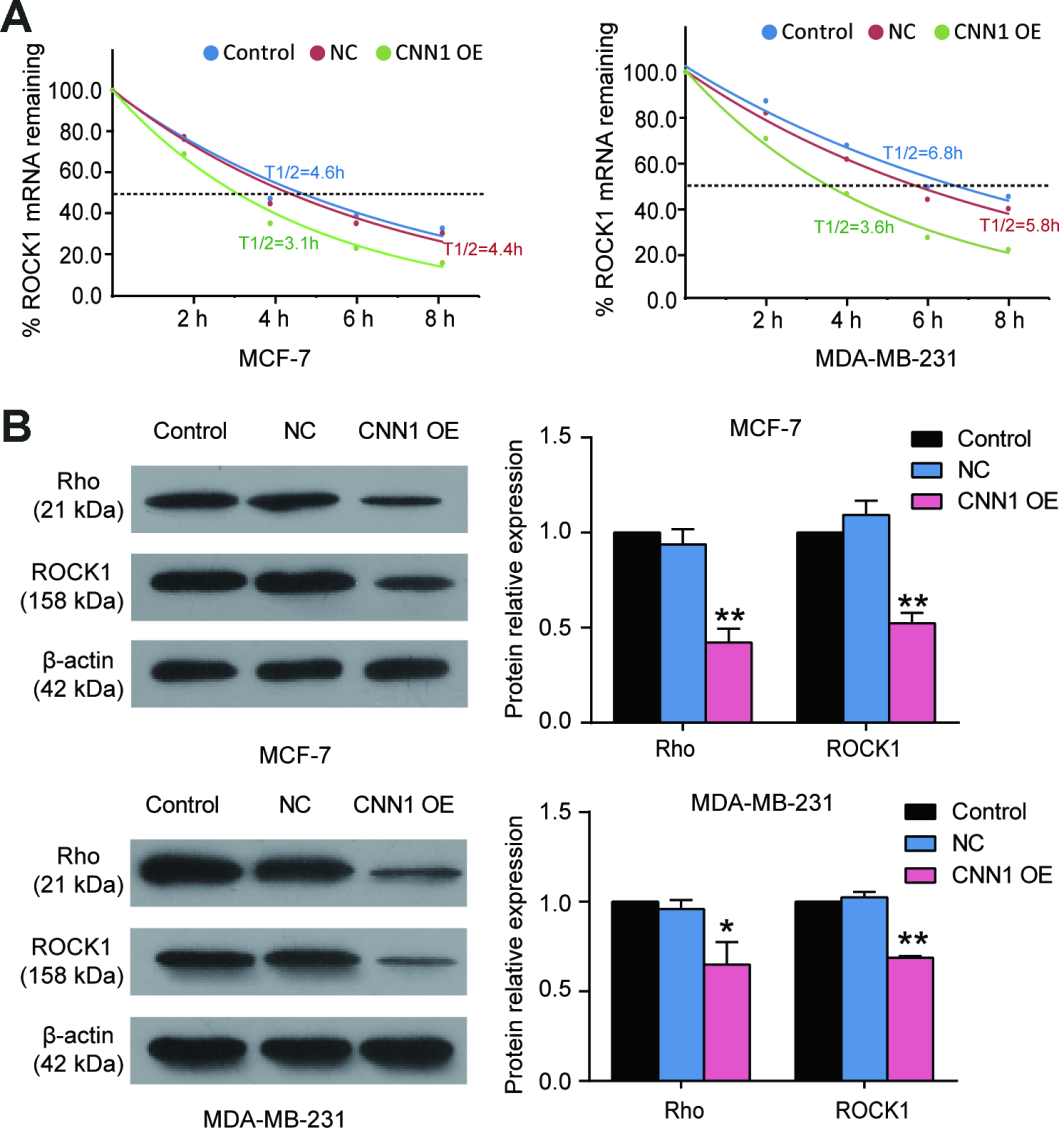
**The Rho/ROCK1 pathway participated in CNN1-induced breast cancer.** (**A**) The half-time of ROCK1 transcript after CNN1 overexpression was decreased using half-time assay. The MCF-7 and MDA-MB-231 cells after transfection with CNN1 overexpression were treated with Act D (8 μg/ml) for 0 h, 2 h, 4 h, 6 h, and 8 h. The ROCK1 mRNA remaining was detected by qRT-PCR. (**B**) The protein expressions of Rho and ROCK1 was decreased after CNN1 overexpression. The western blot assay was used to measure the protein expression after CNN1 overexpression for 24 h. Control, the cells were cultured without any treatment. NC, the cells were treated with the negative control. CNN1 OE, the cells were treated with CNN1 overexpression. *P<0.05 vs. Control and **P<0.001 vs. Control.

To prove Rho/ROCK1 pathway participated in CNN1-induced BRCA process, we first performed half-time assay to detect the ROCK1 mRNA remaining after the BRCA cells transfected with CNN1 overexpression and treated with Act D (8 μg/ml) for 0 h, 2 h, 4 h, 6 h, and 8 h. As shown in Figure 6A, the half-time of ROCK1 transcript in MCF-7 cells was decreased from 4.4 h to 3.1 h after CNN1 overexpression, and the half-time of ROCK1 transcript in MDA-MB-231 cells was also reduced from 6.8 h to 3.6 h after CNN1 overexpression. Then western blot assay was performed, and the result showed the protein levels of Rho and ROCK1 decreased by approximatively 40% when forced CNN1 upregulation was imposed to the cells (Figure 6B). These data indicated that the negative effect of CNN1 on BRCA cells was associated with the suppression of the Rho/ROCK1 pathway.

### miR-106b-5p promoted cell proliferation and lung metastasis through directly targeting CNN1

**Figure 7:**
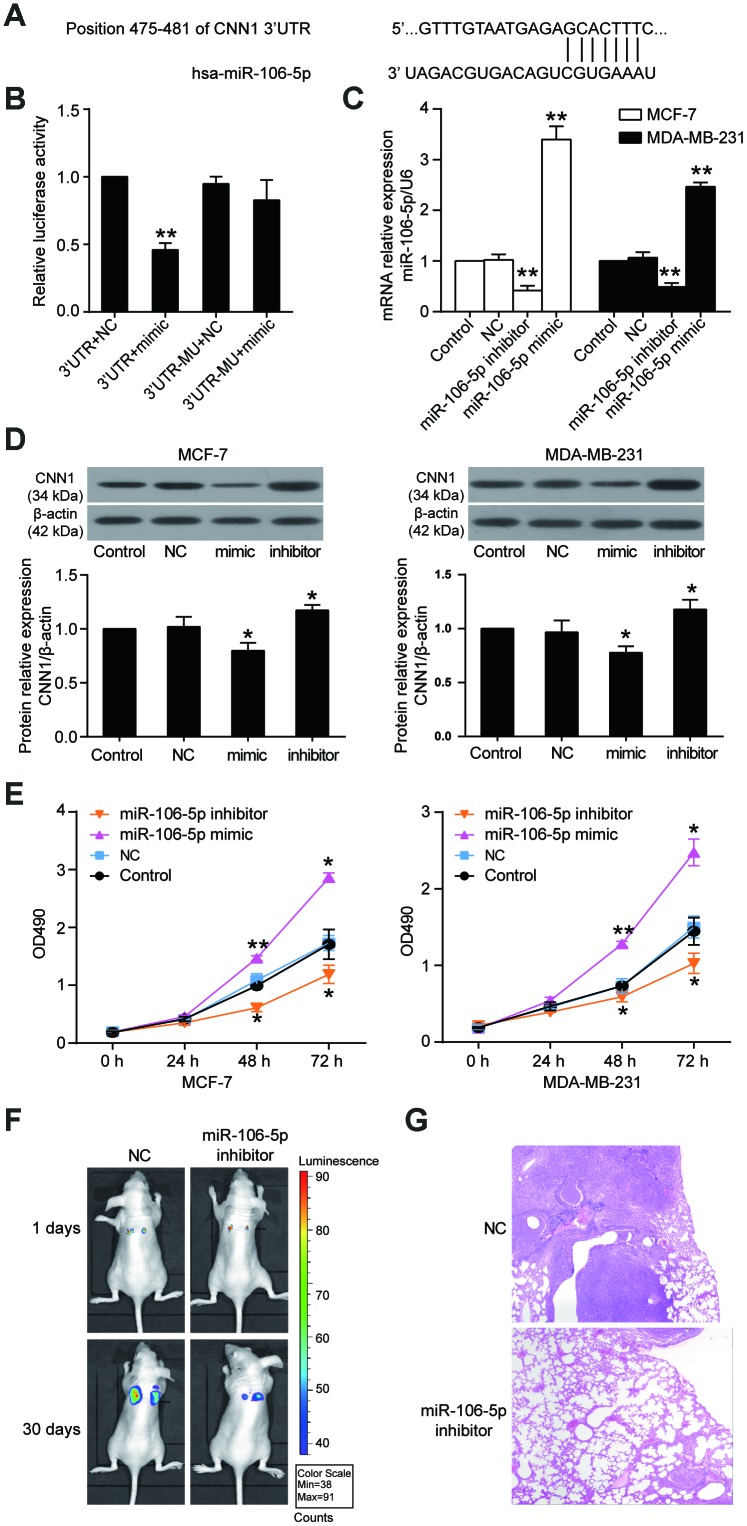
**CNN1 was the target gene of miR-106b-5p, and the miR-106b-5p promoted cell proliferation.** (**A**) The CNN1 3’UTR contained a binding site of miR-106b-5p by miRDB prediction. (**B**) The dual-luciferase reporter assay revealed the interaction between CNN1 3’UTR and miR-106b-5p. The HEK293 cells were co-transfected with the miR-106b-5p mimic and CNN1, or miR-106b-5p mimic and mutated CNN1. 3’UTR, wild-type CNN1 containing 3’UTR binding site. mimic, miR-106b-5p mimic. MU, mutated CNN1 without the 3’UTR binding site. NC, negative control. **P<0.001 vs. 3’UTR+NC. (**C**) The qRT-PCR was performed to detect the transfection efficiency of miR-106b-5p mimic and inhibitor in MCF-7 and MDA-MB-231 cells. (**D**) The miR-106b-5p mimic successfully inhibited CNN1 expression, while the miR-106b-5p inhibitor successfully upregulated CNN1 expression. The CNN1 protein expression was examined by immunoblotting assay after upregulation or downregulation of miR-106b-5p. mimic, the cells were transfected with miR-106b-5p mimic. inhibitor, the cells were transfected with miR-106b-5p inhibitor. (**E**) The miR-106b-5p mimic enhanced cell proliferation, and the miR-106b-5p inhibitor suppressed cell proliferation. The CCK8 assay was performed to detect the cell proliferation after transfection of miR-106b-5p mimic or inhibitor for 0 h, 24 h, 48 h, and 72 h. *P<0.05 vs. Control and **P<0.001 vs. Control. (**F**) Representative live bioluminescence images from mice treated with MCF-7 cells transfected with negative control or miR-106b-5p inhibitor. (**G**) Representative hematoxylin and eosin (H&E)-stained lung sections from mice treated with MCF-7 cells transfected with negative control or miR-106b-5p inhibitor.

Three databases (starBase, miRDB, TargetScan) were used to predict the miRNAs that could bind to the CNN1 3’UTR. 10 overlapping miRNAs were screened out (Supplementary Figure 2), and miR-106b-5p was upregulated in breast cancer but its mechanism had not been investigated before. Therefore, miR-106b-5p was chosen as the miRNA of interest to study in breast cancer. The CNN1 3’UTR contained the binding site of miR-106b-5p according to the prediction of miRDB bioinformatics software (Figure 7A), and the dual-luciferase reporter assay further proved the interaction between CNN1 3’UTR and miR-106b-5p (Figure 7B). The relative luciferase activity was significant reduced when the HEK293 cells were co-transfected with CNN1 3’UTR and miR-106b-5p mimic. As shown in Figure 7C and Figure 7D, the miR-106b-5p mimic successfully transfected into the BRCA cells could lead to a decrease of CNN1 protein level, while the miR-106b-5p inhibitor led to a significant increase of CNN1 protein level. Transfection with miR-106b-5p mimic for 48 h and 72 h, the cell proliferation was enhanced in two BRCA cell lines (Figure 7E). On the contrary, the transfection of miR-105-5p inhibitor caused a decrease at 48 h and 72 h in two BRCA cell lines. *In vivo*, tail vein injection of MCF-7 cells transfected with miR-106b-5p inhibitor resulted in the inhibition of lung metastasis (Figure 7F, 7G). These results suggested miR-106b-5p could promote cell proliferation and lung metastasis by targeting CNN1 in BRCA cells.

### miR-106b-5p alleviated the effects of CNN1 on BRCA cells via the Rho/ROCK1 pathway

**Figure 8:**
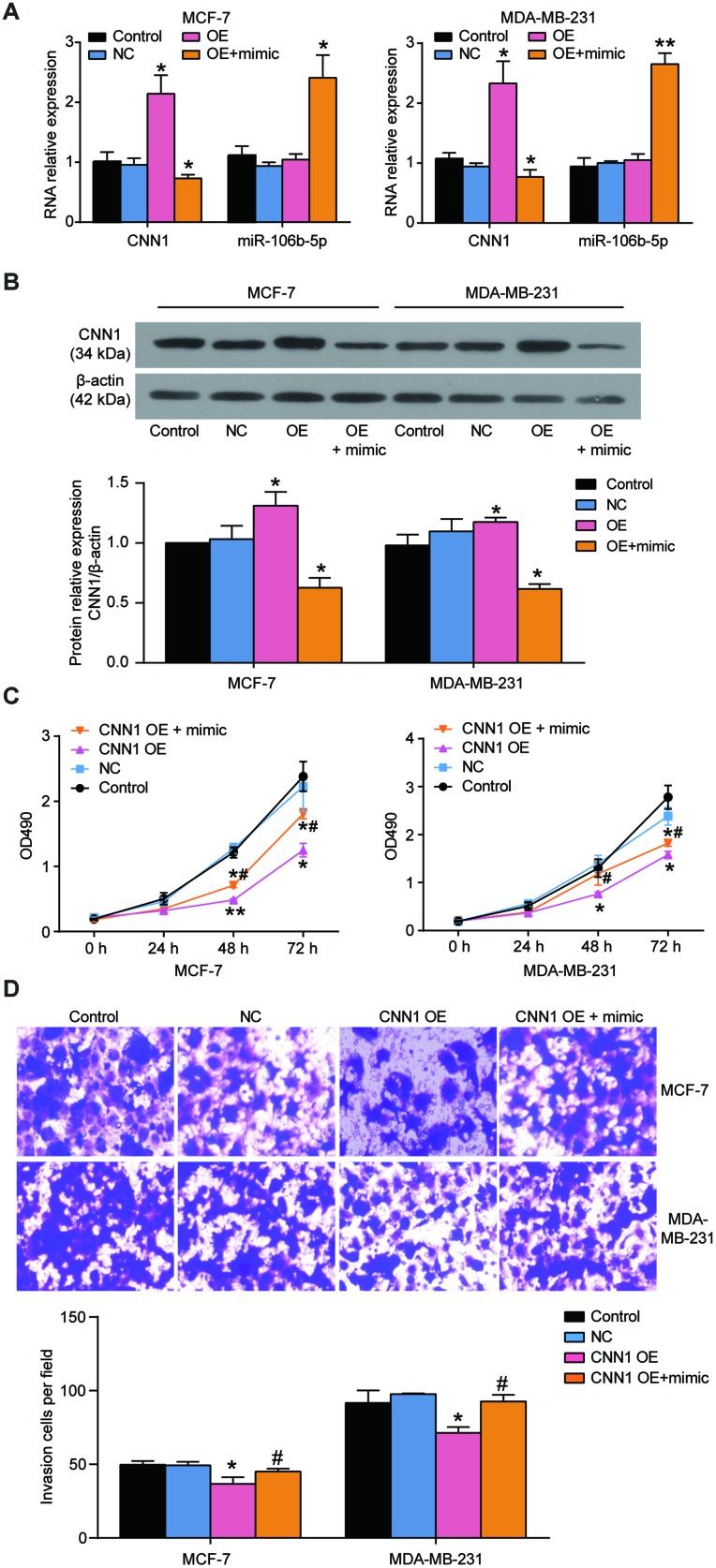
**miR-106b-5p mimic alleviated the role of CNN1 overexpression induced suppression of cell proliferation and invasion.** (**A**) Co-transfection of miR-106b-5p mimic and CNN1 overexpression led to the downregulation of CNN1 and upregulation of miR-106b-5p both in MCF-7 and MDA-MB-231 cells. (**B**) Co-transfection of miR-106b-5p mimic and CNN1 overexpression resulted in the decrease of the protein level of CNN1 compared with transfection of CNN1 overexpression. OE, the cells were transfected with CNN1 overexpression. OE+mimic, the cells were co-transfected with miR-106b-5p mimic and CNN1 overexpression. *P<0.05 vs. Control and **P<0.001 vs. Control. (**C**) and (**D**) The co-transfection of miR-106b-5p mimic and CNN1 overexpression alleviated the inhibition of cell proliferation and invasion caused by CNN1 overexpression. The CCK8 assay and transwell assay respectively revealed the changes of cell proliferation and invasion. CNN1 OE, the cells were transfected with CNN1 overexpression. CNN1 OE+mimic, the cells were co-transfected with miR-106b-5p mimic and CNN1 overexpression. *P<0.05 vs. Control and **P<0.001 vs. Control. #P<0.05 vs. CNN1 OE. #P<0.001 vs. CNN1 OE.

**Figure 9:**
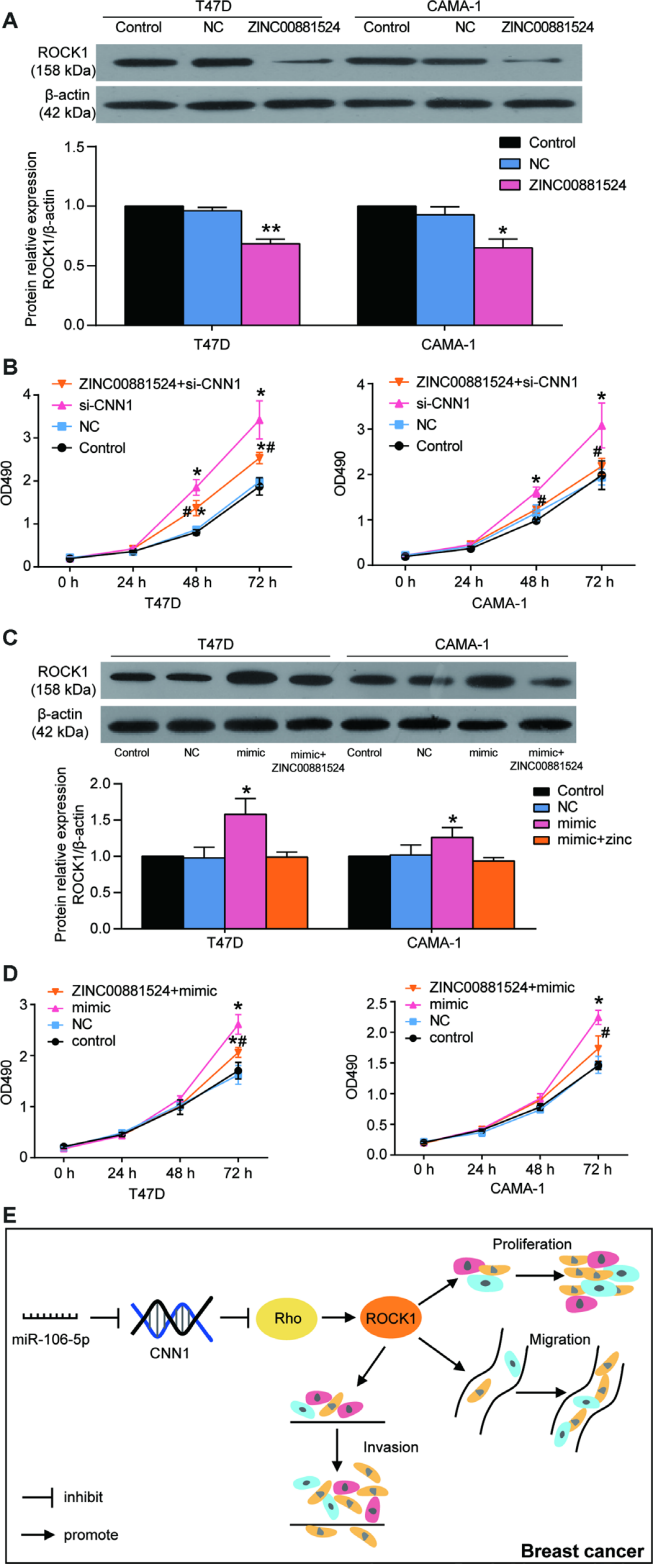
**miR-106b-5p promoted the cell proliferation in T47D and CAMA-1 cells by targeting CNN1 and activating the Rho/ROCK1 signaling pathway.** (**A**) The ZINC00881524 was successfully inhibited the protein expression of ROCK1 after the cells were treated with ZINC00881524 for 24 h. ZINC00881524 is the inhibitor of the Rho/ROCK1 pathway. The protein expression of ROCK1 was detected by immunoblotting assay. (**B**) The CCK8 assay demonstrated that Rho/ROCK1 inhibitor alleviated the cell proliferation compared with the treatment of CNN1 siRNA. si-CNN1, the cells were transfected with CNN1 siRNA. ZINC00881524+si-CNN1, the cells were co-transfected with ZINC00881524 and CNN1 siRNA. *P<0.05 vs. Control and **P<0.001 vs. Control. #P<0.05 vs. si-CNN1. #P<0.001 vs. si-CNN1. (**C**) The protein expression of ROCK1 was upregulated by transfection of miR-106b-5p mimic, but co-transfection of miR-106b-5p and ZINC00881524 could downregulate it. mimic, the cells were transfected with miR-106b-5p mimic. mimic+ ZINC00881524, the cells were co-transfected with miR-106b-5p mimic and ZINC00881524. *P<0.05 vs. Control and **P<0.001 vs. Control. (**D**) Co-transfection of miR-106b-5p and ZINC00881524 alleviated the positive effect of miR-106b-5p mimic on cell proliferation in T47D and CAMA-1 cells. mimic, the cells were transfected with miR-106b-5p mimic. mimic+ ZINC00881524, the cells were co-transfected with miR-106b-5p mimic and ZINC00881524. *P<0.05 vs. Control and **P<0.001 vs. Control. #P<0.05 vs. mimic. #P<0.001 vs. mimic. (**E**) The signaling cascade indicated that miR-106b-5p promoted breast cancer by targeting CNN1 and activating the Rho/ROCK1 signaling pathway.

The MCF-7 and MDA-MB-231 cells were exposed to the miR-106b-5p mimic and CNN1 overexpression confirming that miR-106b-5p could alleviate the effects of CNN1 on BRCA cells through CCK8 assay and transwell assay. Firstly, qRT-PCR results identified that co-transfection of CNN1 overexpression plasmids and miR-106b-5p mimic significantly upregulated miR-106b-5p expression and downregulated CNN1 expression in MCF-7 and MDA-MB-231 cells, whilst the overexpression of CNN1 alone did not affect the expression of miR-106b-5p (Figure 8A). Compared to the cells transfected with CNN1 overexpression plasmids, the protein level of CNN1 was downregulated by almost 50% in both MCF-7 and MDA-MB-231 cell lines co-transfected with CNN1 overexpression plasmids and miR-106b-5p mimic (Figure 8B). The CCK8 assay confirmed that the cell proliferation ability in co-transfection group was increased obviously compared with CNN1 overexpression group (Figure 8C). Similarly, the cell invasion ability of the co-transfected cells also was increased compared with the CNN1 overexpressed cells (Figure 8D). ZINC00881524, the inhibitor of ROCK, successfully decreased the protein level of ROCK1 in T47D and CAMA-1 cells (Figure 9A). CNN1 knockdown together with the treatment with ZINC00881524 resulted in approximatively 30% decrease of cell proliferation in T47D and CAMA-1 cells compared with the CNN1 knockdown group at 72 h (Figure 9B). Besides, the protein level of ROCK1 increased by transfection of miR-106b-5p mimic, but it could be downregulated by co-transfection with ZINC00881524 (Figure 9C). miR-106b-5p mimic transfection together with the treatment with ZINC00881524 led to almost 20% decrease of cell in T47D and CAMA-1 cells compared with the miR-106b-5p mimic-transfected cells (Figure 9D). All the results proved that miR-106b-5p could alleviate the suppression of cell proliferation and invasion in BRCA cells caused by CNN1 overexpression through the Rho/ROCK1 pathway (Figure 9E).

## DISCUSSION

The bioinformatics analysis revealed that CNN1 gene might be a critical gene involving in the development of BRCA. Compared with healthy breast tissues and cells, CNN1 expression was proved to be reduced in BRCA tissues and cells based on the results from qRT-PCR and immunoblotting assays. Forced upregulation of CNN1 expression in BRCA cells suppressed cell proliferation, migration, invasion, and adhesion, whilst enhanced the capacities of cell apoptosis. The half-life of ROCK1 mRNA and protein expression of Rho and ROCK1 decreased after CNN1 overexpression, suggesting that the CNN1 affected the BRCA cells via changing the activity of the Rho/ROCK1 pathway. Besides, miR-106b-5p promoted BRCA cell proliferation *in vitro* and lung metastasis *in vivo*. Our data also displayed that miR-106b-5p mimic weakened the bio-effects of CNN1 overexpression in BRCA cells. The inhibition of the Rho/ROCK1 pathway by ZINC00881524 in cells with CNN1 knockdown or miR-106b-5p mimic confirmed the effect of Rho/ROCK1 pathway in BRCA.

CNN1, is involved in both smooth muscle contraction [26] and the progression of tumors [27]. In the study of Wang et al. [28], downregulation of CNN1 negatively regulated the early metastasis in high-grade serous ovarian carcinoma, and enhanced the ability of cell invasion of ovarian cancer cells. Takeoka et al. [29] found that CNN1 overexpression in human fibrosarcoma cells exhibited a significant decrease in cell motility. Interestingly, Ji et al. reported that CNN2 was significantly upregulated in BRCA than in the healthy [25]. Whether CNN1 is an oncogene like CNN2 or a tumor suppressor gene in breast remains unknown. In our study, CNN1 was found down-regulated in BRCA tissues and cells compared with the healthy breast tissues and cells for the first time. Cellular experiments proved that CNN1 overexpression suppressed breast cancer cell cancerization. Our data revealed that CNN1 might be a potential tumor suppressor gene in BRCA.

In recent years, miRNAs have attracted attention in regulating the progression of breast cancer. For example, miR-218 was found to negatively regulate the zinc finger transcription factor (ZFX), therefore suppressing triple-negative breast cancer progression [30]. miR-665 was found to promote MCF-7 cell proliferation, migration, and invasion, as well as tumor growth and metastasis in xenograft mice [31]. miR-106b-5p was reported to be downregulated in non-small cell lung cancer and its forced upregulation increased the sensitivity of lung cancer cells to cisplatin by directly targeting PKD2 [32]. Haakensen et al. found the miR-106b-5p expression in ductal carcinoma *in situ* (DCIS) and invasive ductal carcinoma (IDC) was significant upregulated, suggesting that miR-106b-5p might promote the development of DCIS and IDC [33]. Lee et al. [13] found that miR-106b-5p was upregulated, and could lead to early breast cancer carcinogenesis by suppressing TGF-β activity. The bio-effects of miR-106b-5p on breast cancer cell canceration was not investigated in the study of Lee et al., although they proved that miR-106b-5p was significantly upregulated in MCF-7 cell line. Then, we confirmed that miR-106b-5p was significantly upregulated in BRCA cells. Forced miR-106b-5p downregulation led to the inhibition of lung metastasis *in vivo*. Forced miR-106b-5p overexpression promoted BRCA cell canceration, and inhibited CNN1 expression *in vitro*. In addition, exogenous miR-106b-5p alleviated the BRCA-inhibition effects of forced CNN1 overexpression.

ROCK1 is a member of the AGC serine/threonine kinase family, and acts as a crucial role in cell migration, cell motility, cell proliferation, and apoptosis [34, 35]. The upregulation of ROCK1 was found in the cancer tissues of BRCA patients, which was associated with poor prognosis outcome [36]. Masahiro et al. [37] had proved that the Rho/ROCK1 pathway was activated in breast cancer cells that led to cell growth. In normal cells and tissues, several CNN1 actin-binding site candidates had been proved to be the substrates of Rho kinase [38]. Moreover, Takeoka et al. [29] found that CNN1 containing the CH domain could inhibit the Rho/Rac pathway to suppress tumorigenicity in human fibrosarcoma cells. Therefore, we speculated that CNN1 overexpression might inhibit the metastasis of breast cancer by suppressing Rho/ROCK1 pathway. Our results showed that forced CNN1 overexpression led to decreased half-time of ROCK1 mRNA. At the same time, the western blot assay showed that both ROCK1 and Rho proteins expression were decreased in CNN1 overexpressed cells. To confirm the mechanism of CNN1 on breast cancer, the inhibitor of the Rho/ROCK1 pathway was used to treat the breast cancer cells. Finally, we found that CNN1 inhibited cell proliferation by suppressing the Rho/ROCK1 pathway. At the same time, the positive effect of miR-106b-5p on breast cancer also was alleviated by suppressing the Rho/ROCK1 pathway.

## CONCLUSIONS

In conclusion, this study reported CNN1 as a tumor suppressor gene in BRCA, since forced CNN1 overexpression inhibited the abilities of cell survival, migration, invasion, and adhesion, but enhanced cell apoptosis. Rho/ROCK1 pathway inhibitor effectively alleviated the role of CNN1 in BRCA cell proliferation and invasion, indicating that miR-106b-5p could promote breast cancer cell cancerization by targeting CNN1 and Rho/ROCK1 pathway. Therefore, we proposed that CNN1 could be a promising therapeutic target for BRCA.

## MATERIALS AND METHODS

### Bioinformatics analysis

The GEO (Gene Expression Omnibus) datasets of GSE1244646 and GSE71053 were downloaded from NCBI (https://www.ncbi.nlm.nih.gov/) with the “breast cancer”, “BRCA” and “Homo sapiens” as the keywords. The GSE124646 included ten healthy breast tissue specimens and ten BRCA tissue specimens, and the GSE71053 included three healthy breast tissue specimens and three BRCA tissue specimens before surgery. Cancer RNA-Seq Nexus (CRN, http://syslab4.nchu.edu.tw/CRN) was a comprehensive online database of cancers. The differentially expressed genes (DEGs) of BRCA were downloaded from CRN containing 42 primary breast tumors samples and 21 normal breast tissue samples. Then the common DEGs from three datasets were obtained using VENNY 2.1 (https://bioinfogp.cnb.csic.es/tools/venny/). Then the common DEGs were uploaded to UALCAN (http://ualcan.path.uab.edu/index.html) to analyze the gene expression in breast cancer samples (n=1097) and normal breast samples (n=114). The Metascape (http://metascape.org/) and String (https://string-db.org/) were performed to construct the co-expression network, and analyze the Gene Ontology (GO) enrichment and Kyoto Encyclopedia of Genes and Genomes (KEGG) enrichment of the common DEGs. Breast Cancer Gene-Expression Miner v4.4 (http://bcgenex.centregauducheau.fr/BC-GEM/GEM-requete.php) is a statistical mining tool to analyze different breast cancer subtypes’ gene expression.

### Tissue specimens and cell culture

The human tissue specimens including healthy breast tissue specimens (n=20) and BRCA tissue specimens (n=20) were obtained from the authors’ institution and approved by the ethics committee. The clinicopathological parameters from 20 BRCA patients were detailed in Supplementary Table 1. The human healthy breast cell line (MCF-10A), four human BRCA cell lines (MCF-7, MDA-MB-231, CAMA-1, and T47D), and HEK293 cell line were purchased from ATCC (USA). The MCF-7, MDA-MB-231, CAMA-1, and HEK293 cells were cultured using DMEM medium (Hyclone, USA), while the T47D cells were cultured using RPMI-1640 medium (Life Technologies, USA). The growth culture medium for cells was prepared by addition of 10% fetal bovine serum (FBS, Hyclone, USA). The six cell lines were cultured at 37°C in an incubator maintaining 5% CO_2_.

### Cell transfection and inhibitor treatment

The lentivirus-mediated CNN1 overexpression vector (CNN1 overexpression), siRNA against CNN1 (si-CNN1, 5'-GCACACAACTACTACAATTCC-3'), miR-106b-5p mimic, miR-106b-5p inhibitor, and negative control were designed, synthesized and obtained from GenePharma Co, Ltd. (China). ZINC00881524, the inhibitor of the Rho/ROCK1 pathway, was obtained from Selleck Chemicals (USA). The 50 nM CNN1 overexpression was respectively transfected into MCF-7 and MDA-MB-231 cells according to the manufacture's instruction, and 50 nM si-CNN1 was respectively transfected into T47D and CAMA-1 cells according to the manufacture's instruction. Besides, the T47D and CAMA-1 cells were also treated with ZINC00881524 to identify the influence of the Rho/ROCK1 pathway.

### The detection of CNN1 gene expression using qRT-PCR

Total RNA for cDNA synthesis was isolated from 20 healthy breast tissues, 20 breast cancer tissues, and five cell lines using TRIzol reagent (Invitrogen, USA). Then 1 μg total RNA for each sample was used to synthesize cDNA using Qiagen One-Step RT-PCR kit (Qiagen Gmbh, Germany). The cDNA, SYBR-Green Master Mix (Applied Biosystems, USA) and primers were mixed to perform qRT-PCR. GAPDH, as the housekeeping gene, was used to normalize the relative mRNA expression. The primer sequences of GADPH were F: 5’-ACAGTCAGCCGCATCTTCTT-3’ and R: 5’-AAATGAGCCCCAGCCTTCTC-3’. The primer sequences of CNN1 were F: 5’-AGGTTAAGAACAAGCTGGCCC-3’ and R: 5’-GCGTACTTCACTCCCACGTT-3’.

### The detection of CNN1 protein level using the immunoblotting assay

The total protein from cells were lysed by RIPA lysis buffer (Beyotime, China) at 4 °C, and centrifuged at 12,000 rpm. BCA Protein Assay Kit (Beyotime, China) was used to detect the protein concentration of the collected supernatant. Next, the total protein was separated using 12% or 5% SDS-PAGE based on the molecular weight of each targeted protein. The separated proteins after SDS-PAGE were transferred and blocked to the PVDF membranes (Millipore, USA) using 5% fat-free milk diluted in TBST buffer. The primary antibodies against CNN1 (Cat#: 46794, Abcam, UK), β-actin (Cat#: ab8226, Abcam, UK), Rho (Cat#: ab40673, Abcam, UK), and ROCK1 (Cat#: ab45171, Abcam, UK) were used to incubate the blocked PVDF membranes at 4 °C overnight. Next day, the membranes were incubated with the second antibody for 2 h. The signal of protein blotting was enhanced by ECL substrate (Millipore, USA). The β-actin as an endogenous protein was used for normalization.

### Cell proliferation and invasion assays

After transfection, the cells were seeded into a 96-well plate with 2000 cells/well. The 10 μL CCK8 solution (Dojindo, Japan) was added to each well, and the optical density value at 0 h, 24 h, 48 h, and 72 h was detected at 450 nm using the Varioskan LUX Multimode Microplate Reader (ThermoFisher Scientific, USA). As for the detection of cell invasion, the 24-well transwell were prepared by adding matrigel (BD Biosciences, USA) to the upper chamber of transwell. The MCF-7 and MDA-MB-231 cells based on the density of 2×10^4^ cells/chamber, and T47D and CAMA-1 cells based on the density of 1×10^4^ cells/chamber were respectively plated into the upper chamber of transwell, and cultured with serum-free medium and 10 μg/mL mitomycin C (Sigma-Aldrich, China). Mitomycin C was added to the cell culture 2 h prior to experiments. The bottom chamber of transwell contained the medium with 10% FBS. After incubation for 24 h, the non-invaded cells were removed, while the invaded cells adhered to the lower surface of the inserts were fixed using 4% methanol for 0.5 h, and stained using crystal violet (0.1%) for 15 min. The images from three random fields were captured to calculate the numbers of invasion cells.

### Assessing the capacities of cell migration and colony formation

The cell migration rate was calculated using wound healing assay. After transfection, the cells (3000 cells/well) were seeded into the 6-well plates to grow up to 95% confluence. Before the wound healing assay, the cells were incubated with serum-free media and 10 μg/mL mitomycin C (Sigma-Aldrich, China). Mitomycin C was added to the cell culture 2 h prior to experiments. Then a sterile plastic tip was used to scratch the cell layer. Washing away the injured cells, the cells was continued to incubate with serum-free media. Finally, the wound distance was measured to calculate the migration rate at the 0 h, 24 h, and 48 h. The migration rate was defined as follows:

$$\text{migration}\;\text{rate}=1-\frac{\text{wound}\;\text{width}\;\text{at}\;\text{per}\;\text{metering}\;\text{point}}{\text{wound}\;\text{width}\;\text{at}\;\text{0}\;\text{h}}$$

To assess the capacity of cell colony formation, the transfected cells cultured up to 80% confluence were trypsinized, resuspended, and plated into 6-well plates according to the density of 500 cells per well. After two weeks, the colonies were fixed with methanol (4%) for 30 min, washed with PBS, and stained with crystal violet (0.1%).

### Assessment of cell adhesion

The 10 μg/mL of type IV collagen was used to coat the 96-well plates overnight at 4°C. Then the plates were washed with PBS and incubated with 1% bovine serum albumin (BSA) for 1 h to block the nonspecific binding. The transfected cells up to 80% confluence were collected, resuspended in serum-free medium, cultured into the 96-well plate according to the density of 2000 cells/well at 37 °C under 5% CO_2_ atmosphere for 30 min and 60min. Washing away the non-adherent cells with PBS after incubation, the adherent cells were fixed with dimethyl sulfoxide (Life technologies, USA) overnight at room temperature. Next day, the Varioskan LUX Multimode Microplate Reader (ThermoFisher Scientific, USA) was performed to measure the absorbance at 540 nm.

### Assessment of cell apoptosis using flow cytometer

The transfected cells were plated into 60-mm plates according to the density of 3×10^5^ cells per plate. After incubation for 30 h, the adherent cells were harvested, rinsed with PBS, and resuspended in 0.5 mL binding buffer. Then the cells were incubated with Annexin V-FITC (Biolead, China) and PI (Biolead, China) according to the volume ratio of 1:2 away from light for 20 min. The single-color staining of Annexin V-FITC or PI was presented in Supplementary Figure 1). Finally, Beckman-Coulter CytoFLEX (Beckman, USA) was used to measure the cell apoptosis rate. The cell percentage in the Q3 quadrant represented the early apoptosis, and the cell percentage in the Q2 quadrant represented the late apoptosis.

### Assessment of half-time of ROCK1 mRNA

To assess the half-time of ROCK1 mRNA, the actinomycin D (Act D) as the transcription inhibitor, was added in the cells to terminate transcription of ROCK1. The cells were plated into 6-well plates to grow up to 90% confluence. Then the cells were transfected CNN1 overexpression or negative control as the previous description. The control cells were cultured without any treatment. Next, the cells were treated with 8μg/mL Act D for 0 h, 2 h, 4 h, and 8 h. The total RNA was extracted from MCF-7 and MDA-MB-231 cells, and the ROCK1 mRNA level was detected by qRT-PCR according to the previous description. The primers sequences of ROCK1 were F: 5’- TGGAGCAGAAGTGCAGAACC-3’ and R: 5’- GCTCCAGTTGCAGGGTTAGA-3’. The half-time of ROCK1 mRNA was calculated by SPSS 19.0 software.

### Confirming the relationship between CNN1 and miR-106b-5p

An online bioinformatics software called miRDB (http://mirdb.org/) predicted the CNN1 3’UTR binding site of miR-106b-5p. To confirm the interaction between CNN1 and miR-106b-5p, the dual-luciferase reporter assay was performed. The wild-type CNN1 3’UTR (WT-CNN1, GTTTGTAATGAGAGCACTTTC) and the mutated CNN1 3’UTR (MUT-CNN1, GTTTGTAATGAGAATGACCGC) were obtained from RiboBio Co., Ltd. (China). The WT-CNN1 and MUT-CNN1 were respectively inserted into the pGL4 vector (Promega, USA) containing the firefly luciferase reporter gene. The HEK293 cells were plated into a 24-well plate according to the density of 5 × 10^5^ cells per well. 100 ng of pGL4-WT-CNN1 or pGL4- MUT-CNN1, 100 nM miR-106b-5p mimic or negative control, and renilla vector were co-transfected into the HEK293 cells. The relative luciferase activities were calculated using the dual-luciferase assay system (Promega, USA) after the cells were incubated for 48 h.

### *In vivo* tumor lung metastasis assay

Female BALB/C nude mice obtained from Charles River Labs (China) were randomly divided into negative control group (4 mice) and miR-106b-5p inhibitor group (4 mice). MCF-7 cells transfected with negative control or miR-106b-5p inhibitor were harvested and resuspended in PBS. Then 2×10^5^ transfected MCF-7 cells were injected into the tail vein of female BALB/C mice. The metastasis of tumor in mice was monitored every week within a month using IVIS Spectrum imaging system (PerkinElmer, USA). After 30 days, the mice were killed, and the lung was dissected and fixed in 10% buffered formaldehyde. The lung tissues were then paraffin embedded and stained with hematoxylin and eosin (H&E).

### Statistical analysis

Data in this study were exhibited as mean ± SD from three independent experiments except for clinical data, and analyzed using SPSS 19.0 software. The statistical analysis was performed using student’s *t*-test, and P<0.05 was considered to indicate a statistically significant difference.

## Supplementary Material

Supplementary Figures

Supplementary Table 1
