# Supplementary material for: miR-106b-5p contributes to the lung metastasis of breast cancer via targeting CNN1 and regulating Rho/ROCK1 pathway
Source: Aging (Albany NY). 2020 Jan 27;12(2):1867–87. doi: 10.18632/aging.102719 (PMC7053600; doi:10.18632/aging.102719)
Supplement: Supplementary Figures [file aging-12-102719-s001..pdf]

SUPPLEMENTARY FIGURES

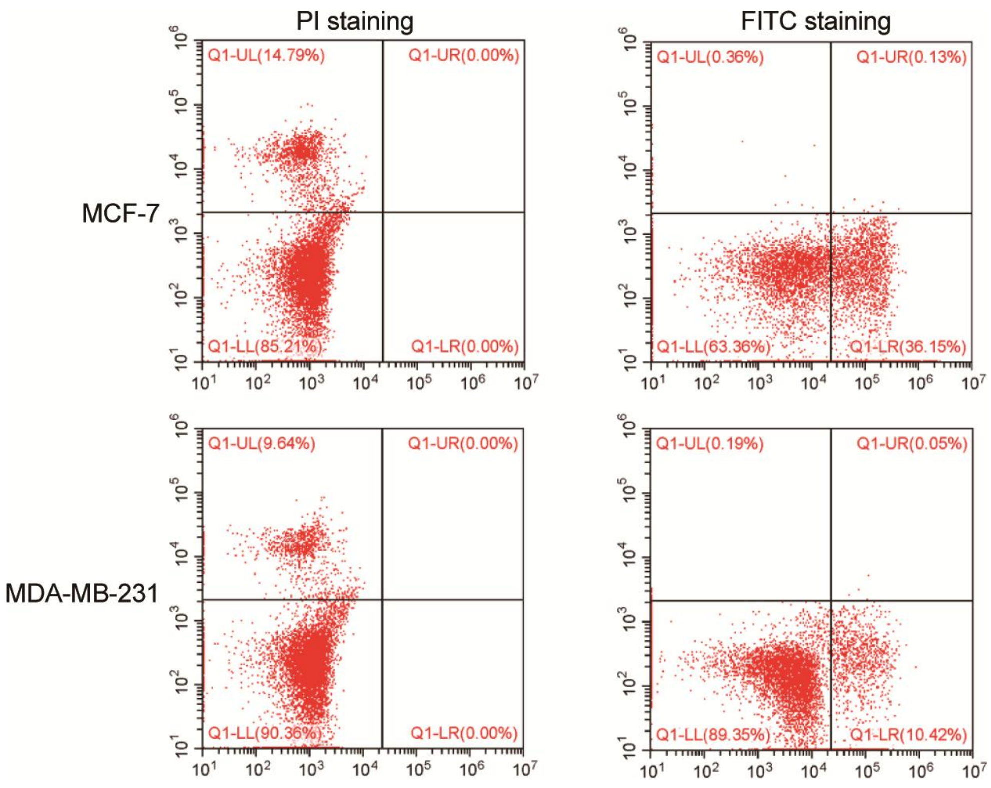

Supplementary Figure 1. Single color staining control for flow analysis of apoptosis.

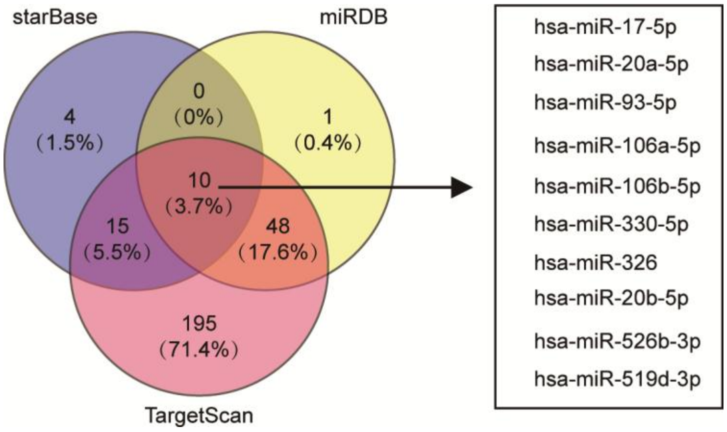

Supplementary Figure 2. Identification of the miRNA of interest. Starbase, miRDB and TargetScan Human 7.2 algorithms were used.
