# Supplementary material for: miR-106b-5p contributes to the lung metastasis of breast cancer via targeting CNN1 and regulating Rho/ROCK1 pathway
Source: Aging (Albany NY). 2020 Jan 27;12(2):1867–87. doi: 10.18632/aging.102719 (PMC7053600; doi:10.18632/aging.102719)
Supplement: Supplementary Table 1 [file aging-12-102719-s002..pdf]

SUPPLEMENTARY TABLE

Supplementary Table 1. The clinicopathological parameters and CNN1 expression of patients with breast cancer.

**Clinicopathological parameters**	**N**	**CNN1**	***P*-value**
Age, year			0.423
<40	9	0.45±0.25	
≥40	11	0.54±0.24	
Tumor size, cm			0.359
<4	7	0.57±0.15	
≥4	13	0.47±0.27	
Histological grade			0.926
Well/intermediate differentiation	10	0.50±0.24	
Poor differentiation	10	0.51±0.26	
Invasion depth			0.002*
Tis-T2	12	0.63±0.19	
T3-T4	8	0.32±0.19	
Lymph node metastasis			0.003*
N0	6	0.73±0.11	
N1-N3	14	0.41±0.22	
Distant metastasis			0.001*
M0	7	0.74±0.09	
M1	13	0.37±0.18	

Tis, tumor *in situ*. * statistically significant.
